# Cryo-EM structure of influenza helical nucleocapsid reveals NP-NP and NP-RNA interactions as a model for the genome encapsidation

**DOI:** 10.1126/sciadv.adj9974

**Published:** 2023-12-15

**Authors:** Florian Chenavier, Leandro F. Estrozi, Jean-Marie Teulon, Eleftherios Zarkadas, Lily-Lorette Freslon, Jean-Luc Pellequer, Rob W. H. Ruigrok, Guy Schoehn, Allison Ballandras-Colas, Thibaut Crépin

**Affiliations:** ^1^ Univ. Grenoble Alpes, CNRS, CEA, IBS, F-38000, Grenoble, France.; ^2^ Univ. Grenoble Alpes, CNRS, CEA, EMBL, ISBG, F-38000, Grenoble, France.

## Abstract

Influenza virus genome encapsidation is essential for the formation of a helical viral ribonucleoprotein (vRNP) complex composed of nucleoproteins (NP), the trimeric polymerase, and the viral genome. Although low-resolution vRNP structures are available, it remains unclear how the viral RNA is encapsidated and how NPs assemble into the helical filament specific of influenza vRNPs. In this study, we established a biological tool, the RNP-like particles assembled from recombinant influenza A virus NP and synthetic RNA, and we present the first subnanometric cryo–electron microscopy structure of the helical NP-RNA complex (8.7 to 5.3 Å). The helical RNP-like structure reveals a parallel double-stranded conformation, allowing the visualization of NP-NP and NP-RNA interactions. The RNA, located at the interface of neighboring NP protomers, interacts with conserved residues previously described as essential for the NP-RNA interaction. The NP undergoes conformational changes to enable RNA binding and helix formation. Together, our findings provide relevant insights for understanding the mechanism for influenza genome encapsidation.

## Teaser

NP-RNA helical assembly reconstituted in vitro provides insights on influenza genome encapsidation by cryo-EM.

## INTRODUCTION

Influenza A virus (IAV) is an infectious respiratory pathogen that continues to be a serious threat by causing seasonal epidemics and occasional pandemics with severe consequences for global public health. Influenza viruses belong to the Orthomyxoviridae family that corresponds to negative-sense, single-stranded, segmented RNA viruses. IAV genome is composed of eight RNA segments assembled into macromolecular complexes called viral ribonucleoproteins (vRNPs) (*1*), which has a central role in viral transcription, replication, and host immunity evasion (*2*–*5*). Within each vRNP, the viral RNA (vRNA) segment is encapsidated by the viral nucleoprotein (NP) and attached to the heterotrimeric RNA-dependent RNA polymerase. Overall, the vRNPs are structured as long flexible helical particles (*6*, *7*) in which the polymerase sits on one end, holding both vRNA extremities, while the opposite end is made of a closing loop. In between, the RNA segment is covered by multiple copies of NP, ordered all along the RNA and forming the nucleocapsid. vRNPs are largely flexible, and thus, all cryo–electron microscopy (cryo-EM) structures available to date are of nanometric resolution (*8*–*10*) and do not allow the visualization of the vRNA or the certain orientation of NP protomers. To overcome this obstacle, we propose an original strategy. Instead of purifying vRNPs from viruses or infected cells, we opted to reconstitute the RNP from recombinant NP protein and short synthetic RNA oligomers. It was known that the viral and recombinant NP protein tends to self-associate as trimers, tetramers, and larger species when purified under physiological conditions (*11*, *12*), which resulted in a dead end for the formation of a helicoidal nucleocapsid structure, even in the presence of an RNA substrate. Therefore, we had established a protocol that allows the purification of the monomeric NP, thus facilitating the formation of helicoidal RNP-like particles (RNP-like) through the binding of a short synthetic RNA (*13*). The RNP-like particles are a tool that mimics the influenza nucleocapsid. Here, we optimized this process, and we used cryo-EM to solve the structure of the reconstituted nucleocapsid or RNP-like to the near-atomic scale. Compared to known vRNP models, this work provides details of the NP-NP and NP-RNA interactions and demonstrates that the NP alone is not sufficient to form the helical structure but requires RNA at the NP-NP interface. On the basis of these results, we propose a mechanism for the assembly of the nucleocapsid along one strand and a model for IAV genome encapsidation within the antiparallel helical vRNP.

## RESULTS

### RNP-like cryo-EM structure

**Fig. 1.**
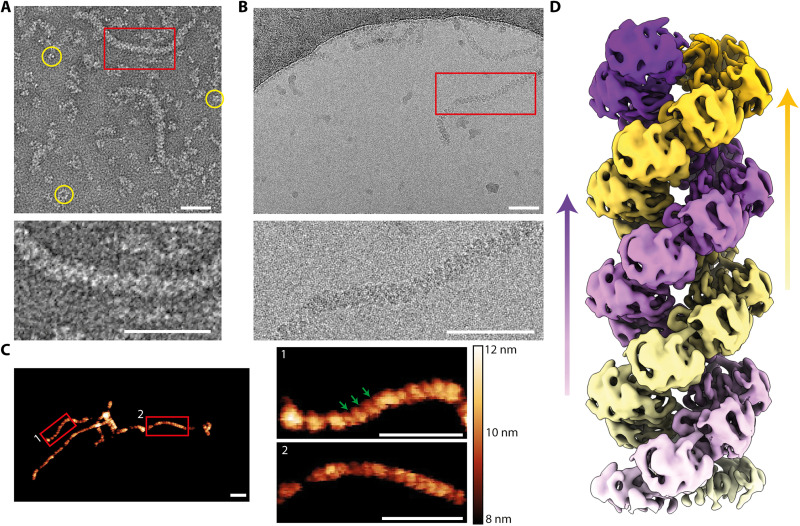
Visualization of the right-handed double-stranded helical RNP-like by EM and AFM. (**A**) EM observation of RNP-like sample by negative staining (sodium silicotungstate). Top, yellow circles highlight the presence of trimers, tetramers, and oligomeric rings of NP. The red boxes [(A) to (C)] correspond to the insets in the neighboring panels. (**B**) Vitrified RNP-like particles in cryo-conditions. (**C**) Observation of the RNP-like particles by AFM. Helical groves are indicated by green arrows. The black-orange-white scale indicates the selected *z* height of the sample, which corresponds to the topography of the particle in nanometers. White horizontal scale bars, 50 nm [(A) to (C)]. (**D**) Helically symmetrized cryo-EM 3D reconstruction of the RNP-like particle at 8.7 Å. The reconstruction displays about two intertwined helical turns. Each strand is colored with either a white-to-purple or a white-to-yellow color gradient. The positive sense of the helical strands is upward indicated by colored arrows.

Our previous work (*13*) has established a protocol that allows the formation of recombinant RNP-like particles, obtained by incubating in vitro purified monomeric influenza A NP with a short synthetic single-stranded ^5^′P-(UC)_6_-fluorescein^3^′ RNA [^5^′P-(UC)_6_-FAM^3^′] overnight. Our former study had compared various poly-UC RNA lengths [5 to 24 nucleotides (nt)] and concluded that 12 nt yields the most RNP-like formation. In addition, we measured the binding of ^5^′P-A_12_-FAM^3^′, ^5^′P-U_12_-FAM^3^′, and ^5^′P-C_12_-FAM^3^′ versus ^5^′P-(UC)_6_-FAM^3^′ to NP using anisotropy fluorescence (table S1), revealing that ^5^′P-(UC)_6_-FAM^3^′ has the best affinity for NP at 150 mM NaCl and thus is the optimal RNA sequence for RNP-like assembly. Inspection of the mixture by negative staining EM revealed helicoidal filaments of nucleocapsid as well as multiple secondary products such as NP trimers, tetramers, and rings (Fig. 1A). A certain level of sample heterogeneity is expected as NP tends to naturally oligomerize in solution (*11*, *12*). Moreover, the absence of the heterotrimeric polymerase to cap the nucleocapsid on one end, coupled with the use of a repeated 12-nt RNA fragment in place of definite length vRNA segments, predictably enables the formation of RNP-like particles of variable lengths. However, although the length of the observed particles may differ from the vRNPs, the 140-Å average diameter measured on negatively stained images concurs with that of the vRNPs (*14*). The high level of RNP-like flexibility is in accordance with the inherent flexibility of the viral RNPs; also, when treated with ribonuclease (RNase), the RNA in both RNP-like and vRNPs is completely degraded (fig. S1, A and B)—as opposed to measles, rabies, and Sendai virus nucleocapsids in which the RNA stays intact (*15*). To establish RNP-like conditions that are most suitable for high-resolution structure determination by cryo-EM, sample optimization was attempted to reduce particle flexibility and increase the particle concentration in the cryo-EM grid holes. Ultimately, pegylation of the sample (*16*) resulted in better particle distribution inside the holes of the grid, slightly reduced flexibility of the RNP-like filaments, and reduced the amount of sample aggregation for cryo-EM analysis (Fig. 1B).

To establish the handedness of the helical assembly, we imaged our recombinant RNPs by atomic force microscopy (AFM) as it allows for the direct observation of surface topography at nanometric scale (Fig. 1C). Close examination of Laplacian-filtered AFM images revealed grooves pointing toward the upper-right in regard to the central axis of the helix, which is characteristic of a right-handed helix conformation. This result is consistent with previous high-speed AFM analysis of vRNP from the PR8 strain, where the authors have also identified a right-handed helix morphology (*17*).

To determine the RNP-like structure, cryo-grids were screened and preliminary images of single particles were acquired using a Talos Glacios 200-kV transmission electron microscope (TEM). The final dataset was acquired on a Titan Krios 300-kV TEM, and the structure was refined using a dataset of 225,892 overlapping segments to an overall resolution of 8.7 Å, which allowed us to achieve the first subnanometric structure of an IAV NP-RNA complex in helical conformation (Fig. 1D, figs. S2 and S3, and table S2). The helical symmetry can be described by two parameters, rise and twist, corresponding to translation/rotation of the asymmetric unit along/around the helical axis, respectively. Each asymmetric unit (NP monomer) is related by a rise of 24.3 Å and a twist of 57.4°, resulting in a right-handed parallel double-stranded helical reconstruction with approximately 6.3-NP protomers per helix turn. Of note, the previous vRNP models were reported as left-handed (*8*, *10*) or right-handed (*9*); however, the handedness cannot be assessed with certainty at the given resolutions by cryo-EM—unless one compares a tilted and untilted image of the same particle. With our cryo-EM data, we undoubtedly fit the crystal structure of IAV NP, notably the pack of α helices of the head domain, in the EM map (fig. S3C). Combined with the AFM data, these results confirm that our RNP-like particles are assembled as a right-handed helix.

### Focused 3D reconstruction and pseudo-atomic model

**Fig. 2.**
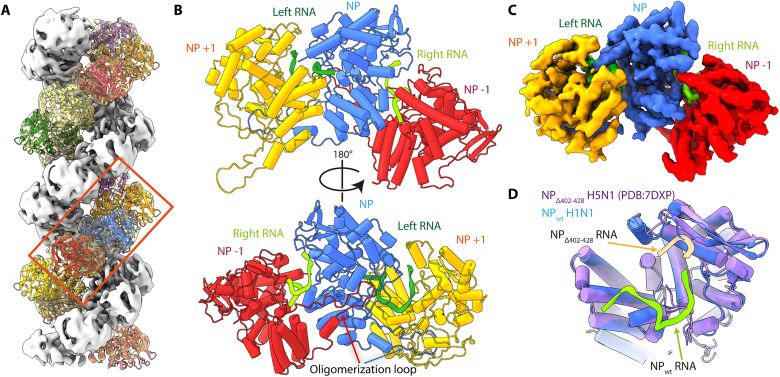
Local reconstruction and NP atomic model. (**A**) Fitting of symmetrized NP atomic model modified from PDB code: 2IQH. Each protomer is colored differently and displayed as cartoon representation. The boxed region corresponds to the area used for focused 3D reconstruction at 5.3-Å resolution. (**B**) Resulting pseudo-atomic model of three neighboring NP protomers, NP^−1^, NP, and NP^+1^ in red, blue, and yellow ribbons, respectively, and two RNA molecules at the NP-NP interface, RNA left and right in green and chartreuse ribbons, respectively. (**C**) Of note, the oligomerization loop of the NP^+1^ protomer is extrapolated on the basis of the oligomerization loop of protomers NP and NP^−1^ as it is not included in the focused reconstruction map. Top, view from the central axis of the helix of the atomic model build from the focused reconstruction map (5.3 Å). Bottom, view toward the center of the helix of the same nucleo-protomers. Oligomerization loops are clearly visible, entering the neighboring NP protomer. Focused 3D reconstruction map colored to corresponding NP protomers, NP^−1^, NP, and NP^+1^ in red, blue, and yellow, respectively. (**D**) Overlay of the central blue NP protomer with chartreuse left RNA, with the crystal structure of IAV H5N1 NP in purple, with three RNA nucleotides in beige (PDB code: 7DXP).

**Fig. 3.**
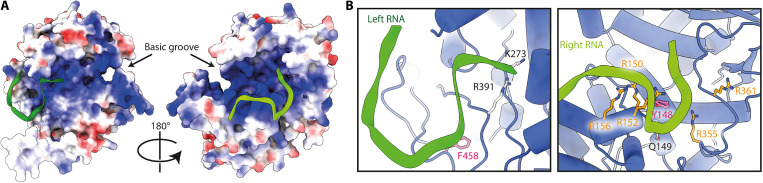
Visualization of the RNA molecules in regard to the NP central basic groove. (**A**) Localization of the RNA molecules on the positively charged region of NP. Electrostatic surface representation of NP colored according to the residues charged positively (blue) or negatively (red). (**B**) Close-up views on the right and left RNAs shown in chartreuse and green ribbons; the NP residues most likely interacting with the RNA phosphate backbone, bases, or in the close vicinity of the RNA are shown respectively as sticks colored in orange, pink, and white, while the rest of the protein appears as blue ribbons.

Although the overall resolution of the map is 8.7 Å, local resolution reaches 6.5 Å toward the central axis of the helix, revealing density regions that are characteristic of α-helix folding, and thus allowing the fitting of the head domain from IAV NP crystal structure (Fig. 2A and fig. S3C). The body domain of NP could be roughly fitted in the remaining EM density; however, precise pathway for the peptide chain remained largely unclear. Therefore, we performed a local refinement (see Materials and Methods) focused on three successive NP protomers along the same strand (called later in the text NP^−1^, NP, and NP^+1^, respectively) (Fig. 2, A, boxed region, and C). This has further increased our focused reconstruction structure resolution to 5.3 Å, with local resolution varying from 8 Å in the periphery of the focused reconstruction structure to 4 Å throughout the core region (fig. S4, A and B). The focused reconstruction structure allowed the unambiguous fitting of three NP protomers and the building of a pseudo-atomic model based on the x-ray crystal structure of A/NP [Protein Data Bank (PDB) code: 5TJW] covering the entire sequence except for the last eight residues at the C-terminal end of NP (fig. S5). The oligomerization loop is clearly visualized, anchored in the neighboring NP subunit (Fig. 2B and fig. S5, A and D). There are two remaining empty densities at the interface between NP protomers, which we were able to assign to RNA (Fig. 2B and fig. S4C). These densities are located adjacent and between the positively charged NP surface (Fig. 3A; NP topology defined in fig. S6). These two densities can roughly accommodate 10 and 12 nt, respectively, between NP^−1^-NP (right RNA) and NP-NP^+1^ (left RNA) and both corresponding to the synthetic 12-nt RNA used for recombinant RNP-like assembly. Superposition with the crystal structure of the monomeric IAV H5N1 NP with three RNA nucleotides (PDB code: 7DXP) revealed that the two RNAs are in line, forming an almost continuous pathway, leading toward the basic grove and ultimately to the subsequent RNA at the NP-NP interface (Fig. 2D). Of note, the RNA density local resolution varies between 4.5 and 6.8 Å, suggesting that the RNA is globally dynamic within the RNP-like structure; therefore, we only modeled the phospho-ribose chain, and we did not assess the direction (5′ to 3′) of the RNA structure.

### RNA binding in the context of the RNP-like

Despite the abundance of studies on the binding between IAV NP and RNA, the exact mechanism of their interaction remains unclear. Here, we observe that residues R150, R152, R156, R355, and R361 most likely interact with the RNA phosphate backbone and that Y148 and F458 may interact with the RNA bases, while residues Q149, K273, and R391 are in the close vicinity of the RNA and could also be involved in its binding (Fig. 3B). All these residues were already highlighted in previous studies either through mutations or reverse genetic generation of viruses as involved in the NP-RNA interaction (recapitulated in table S3) (*18*, *19*). Moreover, sequence alignment of NP sequences from IAV, IBV, ICV, and IDV (fig. S7) reveals that most of these residues are conserved, supporting their important role in influenza viruses biology.

The RNA observed in our EM density agrees with previous NP-RNA interaction studies; nonetheless, no density was observed in the central basic groove as one would expect (fig. S6). The small RNA molecule is located at the interface between two NP protomers. It is possible that an RNA molecule is also present at the central basic groove (made by the hinge between the head and body domains) with low occupancy, and because of data treatment, only the most stable conformation was selected, averaged to give signal, and thus visualized. This strongly supports that the RNA is a major component of the RNP helical assembly and stabilization by bridging the NP-RNA-NP. Our previous research on the assembly of RNP-like particles (*13*) could not explain why helical assembly did not occur using RNA molecules smaller than 10 nt long. Observation of our structure indicates that an RNA molecule of at least 10 nt is required to bridge the NPs together at the NP-NP interface and thus to form the RNP helical architecture. In our hands, the most efficient protocol to form RNP-like particles uses a 12-nt synthetic RNA molecule; as we increase incrementally the length of the RNA up to 36 nt, the yield of RNP-like largely decreases (fig. S1C). We suspect that in our simplified in vitro system, a short RNA molecule promotes the NP oligomerization into RNP-like structure. Longer RNA molecules could affect this assembly by inducing steric hindrance and clashes to access the central basic groove as protein and RNA are mixed together all at once, contrary to the formation of a progeny vRNP in the host cell, where NPs are progressively binding to the newly synthetized RNA as elongation proceeds.

### NP dynamics upon oligomerization into RNP helix

**Fig. 4.**
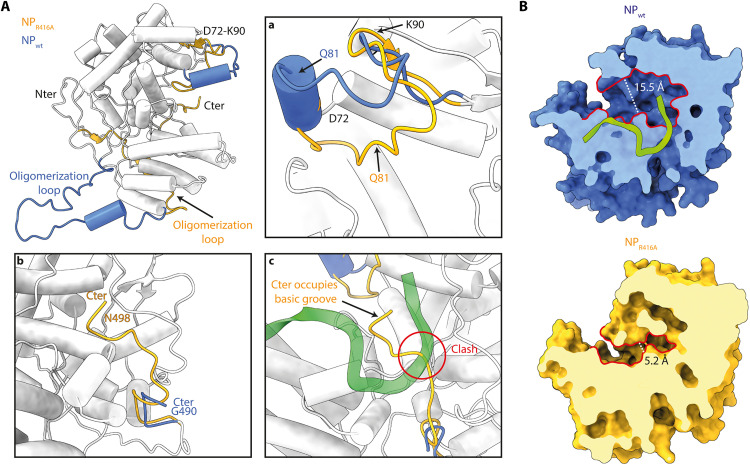
Comparison of the NP conformations from the RNP-like helical structure (NPwt) with the monomeric NP R416A mutant crystal structure (NP_R416A_). (**A**) Superimposition of the NP protomer from the RNP-like reconstruction (NPwt) and the monomeric NP_R416A_ crystal structure (PDB code: 3ZDP). For clarity, RNA was omitted from NPwt. The global root mean square deviation (RMSD) is 0.83 Å, and the two structures are shown as a single chain colored in white, except for when the local RMSD is more than 3 Å; then, NPwt is displayed in blue and NP_R416A_ in yellow. The oligomerization loop in NP_R416A_ is folded inside the protein, while the oligomerization loop of NPwt appears pointing to the solvent. Box (a) corresponds to a close-up view on the D72-K90 loop, either folded in an α helix in NPwt (blue) or unfolded in NP_R416A_ (yellow). Box (b) displays the C-terminal tail (G490-N498) either bound to the rest of the protein in NP_R416A_ (yellow) or not visible in the NPwt EM density—Note that the RNA is omitted in this box. Box (c) shows the steric clash between the RNA molecule from NPwt and the C-terminal residues of NP_R416A_. (**B**) Central basic groove accessibility is modified by disordered D72-K90 loop folding. Surfaces of NPwt (top) and NP_R416A_ (bottom) are presented in the same orientation and colored in blue and yellow, respectively, with the RNA molecule appearing in chartreuse cartoons. Surfaces are sliced up to the beginning of the NP-RNA central basic groove for clarity. Contour of the basic groove entrance is lined in red, and the size of the grove is indicated by the white dotted line.

Numerous structures of the influenza NP have been solved using x-ray crystallography, including variants with mutations such as R416A or in the presence of multiple inhibitors (*20*–*22*). The overall structures are similar, except for the oligomerization loop (residues 402 to 428), which displays considerable flexibility and various orientations, as well as a loop spanning residues D72 to K90, which also revealed several conformations and notably an α helix in our structure (Fig. 4A). The R416A mutation has been shown to disrupt NP oligomerization by eliminating a crucial interaction in the oligomerization pocket, where R416 forms an ionic bond with E339, stabilizing the trimeric conformation. As a result, the oligomerization loop folds on the NP core rather than binding the oligomerization pocket of the NP^+1^ protomer, and consequently, the R416A NP mutant is monomeric and unable to form RNPs. Previous studies (*19*, *23*) highlighted the dynamic nature of the flexible loop D72-K90 and its role with RNA interactions. All x-ray structures revealed multiple conformations of this loop, ranging from being too flexible to be observed (e.g., IAV NP, PDB code: 2IQH; IBV NP, PDB code: 3TJ0; and IDV NP, PDB code: 5N2U chains A and B) to visible, disordered, and pointing toward the NP central basic groove (e.g., IAV NP R416A, PDB code: 3ZDP) or folded into an α helix (e.g., this structure, IAV NP, PDB code: 5TJW; IDV NP, PDB code: 5N2U chains C and D). The large range of possible conformations emphasizes the high level of dynamism in this region. In addition, the residue K87 of this loop was described as susceptible to succinylation; this posttranslational modification was shown to affect vRNP assembly (*24*). Furthermore, the disordered conformation, observed with monomeric R416A mutant, precludes the access to the central basic groove by occupying space (Fig. 4B, bottom), while the folded conformation observed with our structure opens up the cavity and positions the R74 and R75 residue side chains toward the opened cavity (Fig. 4B, top). This observation suggests that the D72-K90 loop plays a regulatory role and enables the RNA access to the central basic groove via dynamic and transient conformational changes.

The NP flexible C terminus is lacking the last eight C-terminal residues in our cryo-EM density as well as in all the x-ray NP structures where oligomerization is enabled (PDB code: 2IQH, 5TJW). In contrast, the C terminus is entirely visible within NP structures where oligomerization was impaired, either by mutation (e.g., R416A) or deletion of the oligomerization loop (PDB code: 7DXP). The C-terminal tail (residues 490 to 498) is then making direct interaction with the residues R150, R152, and R355 at the exact position where RNA density is observed in our RNP-like structure (Fig. 4A and fig. S8) (*19*). Together, these elements suggest that the IAV NP undergoes substantial conformation remodeling to switch from a monomeric NP state to a helical RNP state.

## DISCUSSION

Our study presents the first subnanometric structure of the NP-RNA complex in a helical conformation. We were able to visualize a parallel double-stranded helical assembly at a resolution sufficiently high to describe the conformational changes of NP to accommodate RNA binding and oligomerization into nucleocapsid (movie S1). It is important to point out that our helical reconstruction is straight and does not fully reflect the highly flexible nature and the inherent dynamic of our ribonucleoprotein-like particles and so the vRNP. We used short segments of the RNP-like particles to perform two-dimensional (2D) classification (fig. S2 and table S2), and we selected the best aligned 2D classes, corresponding to the straightest segments, to build a 3D reconstruction. In interpreting our data, we kept in mind the high level of flexibility and dynamism of the sample, which explains that even if the RNA seems completely buried at the NP-NP interface in our structure, it could be accessible to RNases in solution. Our RNP-like structure is thus a biological tool and a working model for the influenza nucleocapsid assembly until the high-resolution structure of a native vRNP is unraveled.

**Fig. 5.**
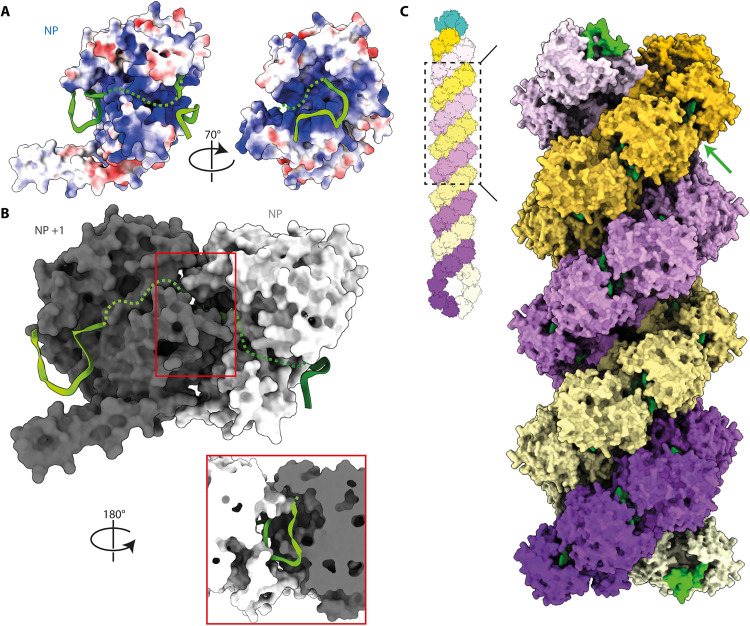
Model of the full pathway of the RNA and schematic model of the vRNP. (**A**) Proposed RNA pathway. Electrostatic surface representation of an NP protomer and two green ribbon RNA molecules as seen from the RNP-like focused reconstruction. The green dotted line corresponds to the proposed RNA pathway. (**B**) Surface representation of two neighboring NP protomers in dark gray and white, visualized from the center of the helix. The structure of the RNA from the cryo-EM structure appears in green ribbon representation, and the green dotted line corresponds to the proposed pathway of the rest of the RNA for each protomer. Boxed region, close up on one RNA molecule enclosed at the NP-NP interface visualized toward the center of the helix. (**C**) Schematic model of a vRNP (top corner); antiparallel helix modeled by symmetry from the RNP-like reconstruction; the RNA-dependent RNA polymerase is represented in sea green. NP protomers from one strand are colored as gradients from light to deep purple, and from light to dark yellow for the second strand. The RNA is colored in green; the arrow indicates a potential area for the formation of an extended loop of RNA.

Notably, the two visible RNA molecules in our density are positioned right next to the central basic groove, and this allows us to model the continuous RNA pathway, across the groove, as shown by the dotted line in Fig. 5 (A and B). With this modeling, about 22 to 24 nt can be fitted per NP protomer (12 nt buried at the NP-NP interface and about 10 to 12 nt across the central basic groove), agreeing with the previous studies establishing the RNA (24 nt) for one NP stoichiometry using mass spectrometry and biochemistry coupled with EM (*25*, *26*).

To propose a picture even closer to that of a vRNP, we generated the antiparallel RNP-like conformation by flipping one strand by 180° around an axis perpendicular to the helical axis without any steric clashes (Fig. 5C). Our model could also accommodate longer RNA loops that would extend from the tip of the U-shape RNA held between two neighboring NPs (Fig. 5, B and C, and fig. S9C). These RNA loops pointing out of the nucleocapsid would allow for intersegment RNA interactions that are necessary for the packaging of the eight segments constituting the IAV genome (*27*, *28*).

In addition, our study sheds light on the divergence between nonsegmented and segmented negative-strand RNA viruses based on their nucleocapsid formation. For nonsegmented negative-strand RNA viruses, the *Mononegavirales*, like measles, rabies, and Ebola viruses for which the atomic structure of the NP bound to vRNA is known, the nucleocapsids are rather straight helices, and each protomer binds between 6 and 9 nt (*29*). However, the RNPs of the segmented viruses, like the *Bunyavirales* (the Bunyaviruses and the Arenaviruses), are very flexible, and the NP protomers bind between 7 and 11 nt (*30*–*33*), while the consensus for IAV, an *Orthomyxoviridae*, agrees on 20 to 26 nt per NP protomer (*7*, *25*, *26*). It also appears that the measles virus prefers binding purines (*34*) and forms a left-handed nucleocapsid helix (*35*). However, the IAV and Bunyaviruses that bind better to pyrimidines, like C and U (*13*, *31*), use RNA to bridge neighboring NP protomers (fig. S9) and assemble into right-handed helices (*17*, *33*). These observations highlight the differences between *Mononegavirales* and *Articulavirales*. Together with our data, this suggests that the RNA is allowed more movement in the IAV RNP than in *Mononegavirales* nucleocapsids (*32*, *36*), explaining the high flexibility of the vRNPs and the resolution limit we achieved with this structure.

We deciphered how the NP oligomerization loop together with the NP regulatory loop, NP C-terminal lock, and RNA bridge are essential to promote nucleocapsid assembly. We did not observe interstrand interactions that would be relevant for vRNP coherence. Known x-ray structures provide snapshots of NP before and after oligomerization and/or RNA interaction that could correspond to putative intermediate states. On the basis of these structures, we hypothesize conformational events that are required for the RNA encapsidation concomitantly to the elongation of the nucleocapsid helix. The comparison of the x-ray structures of monomeric and trimeric NP indicate that the oligomerization induces the displacement of the NP C terminus, unmasking the “R150-R152-R355” site that corresponds to an RNA binding surface. We then propose that as NP docks on the previous protomer by locking the oligomerization loop in the neighboring NP receiver pocket through the well-described NP_R416_-NP_E339_ salt bridging, the RNA can bind to the R150-R152-R355 surface that is now accessible. In addition, upon RNA binding, the flexible D72-K90 loop folds into an α helix, increasing the accessibility of the central basic groove. Together, these observations highlight the inherent dynamic of NP that reflects the high flexibility of the viral RNP and confirm previous works regarding NP dynamics (*20*, *23*) and RNA binding (*19*). Considering also the cellular context of the viral infection, many host factors have been shown to participate in the replication. Specifically, a recent paper shows that IAV NP C-terminal mobility is also affected by the presence of ANP32A (*37*).

Overall, this work presents the RNP-like as a biological tool to study the vRNP biology. Like every model, there are limitations; for one, the reconstituted RNPs form a double parallel helix as opposed to the antiparallel helix of native vRNP, most likely due to the short RNA and the absence of the polymerase. An antiparallel helical model was generated and appears plausible; however, until this antiparallel model is further validated, interactions between opposite strands have not been taken into consideration. The native vRNPs are dynamic and flexible, and therefore, they adopt various states ranging from compacted (*38*, *39*), featuring major and minor grooves (*39*–*41*), to relaxed (*7*, *40*), depending on the buffer conditions and visualization/staining methods. The RNP-like structure resembles the compacted state of vRNP; however, because of the parallel helical assembly, this structure can only provide meaningful information regarding the intra-strand interactions such as the NP-NP oligomerization, NP-RNA interaction, and overall architecture along one strand.

Together, the RNP-like particles should be considered as a relevant tool to investigate how the RNA is encapsidated by the NP and contribute to a better understanding of the molecular mechanisms underlying RNA virus assembly and replication.

## MATERIALS AND METHODS

### NP purification and RNP-like formation

The recombinant NP (A/WSN/1933) purification and RNP-like assembly were performed as previously described by Labaronne *et al.* (*13*).

### Anisotropy fluorescence

Black 384-well plates (BRAND) were used for anisotropy fluorescence measurement. Serial protein dilutions were performed at room temperature using the filtrated elution buffer [20 mM tris-HCl (pH 7.5), 50 mM NaCl, and 5 mM β-mercaptoethanol]. RNA molecules labeled at the 3′ end with 6-fluorescein amidite (FAM) were diluted to 5 nM within 60 μl. RNA addition to NP was performed as the last step before a 10-min incubation. Anisotropy measurements were realized using a Clariostar microplate reader (BMG Labtech). Excitation was performed at 480 nm, and emission was recorded at 520 nm. Data plotting were done with GraphPad Prism after removing anisotropy value of RNA-FAM alone for all data. Dissociation constants (*K*_d_) were calculated using the single binding site with Hill slope (h) function of GraphPad Prism.

### AFM imaging

The 100 μM RNP-like solution was diluted to 0.2 μM in Milli-Q water. The sample solution was deposited on freshly cleaved mica for 2 min and then dried under a stream of nitrogen gas. All imaging was conducted with the PeakForce Tapping mode using the ScanAsyst mode of a multimode 8 microscope and a Nanoscope V controller (Bruker, Santa Barbara, USA). Whenever the ScanAsyst mode is applied, we manually adjust both the set point and gain to optimize imaging. Images were scanned at a rate of ∼1.0 Hz. The HIRS-F-A cantilever was used (nominal *k* = 0.35 N m^−1^, Fq = 165 kHz, and tip radius = 1 nm; Bruker Probes, Camarillo, CA, USA). The image resolution was maintained around 1 nm per pixel, and image correction was performed with Gwyddion version 2.53 (*42*). Further image processing includes noise removal using DeStripe (*43*) and the Laplacian Weight Filter (*44*, *45*).

### Negative stain grid preparation and visualization

A 4-μl drop of freshly assembled RNP-like diluted to 0.05 mg/ml was applied to the clean carbon side of a carbon/mica interface. The sample on carbon was then immediately stained using a 2% (w/v) sodium silicotungstate (pH 7.0), 200-μl drop, fished with a 400-mesh copper grid (Quantifoil), and air-dried at room temperature. Images were collected on a Tecnai F20 electron microscope (FEI Tecnai, Hillsboro, OR, USA) operating at 200 kV at a nominal magnification of ×55,000 and equipped with an FEI Ceta camera.

### Cryo-grid preparation and data collection

Before vitrification, the sample was incubated for 30 min on ice with 2 mM methyl-PEG8–*N*-hydroxysuccinimide ester reagent (Sigma-Aldrich) and then diluted to 0.2 mg/ml in 50 mM Hepes (pH 7.5), 150 mM NaCl, and 2 mM β-mercaptoethanol. Subsequently, a 4-μl drop of the pegylated dilution was deposited onto a Quantifoil holey carbon grid (R0.6/1, 300 mesh, copper) previously glow-discharged for 45 s at 25 mA. The grid was incubated for 10 s at 20°C under 100% humidity using a Mark IV Vitrobot (Thermo Fisher Scientific), blotted for 5 s using force 0, and plunge-frozen in liquid ethane for specimen vitrification. The grids were screened for sample integrity and ice quality on a Talos Glacios 200 kV FEG microscope (Thermo Fisher Scientific) equipped with a K2 summit direct electron detector camera (Gatan).

An initial dataset of 11,413 movies of 40 frames was collected at 1.145 Å per pixel with a dose rate of 1 e^−^/Å^2^ per second. Initial 2D classes revealed a repeated pattern characteristic of helical assembly. An initial model was obtained at 15-Å resolution (fig. S10), which is too low to determine helical parameters even if preliminary values of pitch and twist could be estimated on the basis of the 2D classes. This data collection attests to the sample quality and supports the possibility to achieve higher resolution with more particles.

The images used to refine the RNP-like structure were acquired on a 300-kV Titan Krios microscope (Thermo Fisher Scientific) equipped with a K3 direct electron detector (Gatan) camera functioning in counting mode and coupled with a Quantum-LS energy filter at the ESRF CM01 in Grenoble, France (*46*). A total of 26,515 movies of 40 frames each were collected using EPU (Thermo Fisher Scientific), with a dose rate of 1 e^−^/Å^2^ per frame and a total exposure time of 1.9 s, which corresponds to a total dose of 40 e^−^/Å^2^. Movies were collected at a magnification of ×105,000, corresponding to a calibrated pixel size of 0.84 Å per pixel with a defocus ranging from −0.8 to −2.2 μm.

### Cryo-EM image processing and structure refinement

The movie frames were motion-corrected and dose-weighted using MotionCor2 (*47*). The motion-corrected micrographs were imported into CryoSPARC v3.3.2 (*48*), which was then used to perform patch contrast transfer function estimation and micrograph manual curation. Initial 2D templates were generated on the basis of manually picked subset of straight filaments with filament tracer by using a diameter of 150 Å, minimum length of 300 Å, and filament segmentation every 24 Å. The resulting picked segments were first manually inspected and extracted within 440 × 440 pixels^2^ box before discarding flexible segments via several rounds of 2D classification. An ab initio initial 3D model was generated using CryoSPARC before 3D refinement and before determination of helical symmetry parameters. These parameters (rise of 24.3 Å and twist of 57.4°) were applied during a 3D helical refinement job to achieve an 8.7-Å resolution from 225,892 segments.

Particle orientations and coordinates were then exported to RELION v4.0 (*49*–*51*) for further processing. The particles were subjected to 3D refinement with the CryoSPARC final model as reference (low-pass filter at 30 Å) and unsupervised 3D classification into four classes with and without image alignment. The resulting classes were selected, and a final helical refinement was performed for each of them, reaching the same resolution (8.7 Å) as CryoSPARC. The determination of helical parameters in CryoSPARC displayed a broad local minima, corresponding to small variations of rise and twist, which explains the middle-low resolution of each helical refinement. Thus, to achieve higher resolution, signal subtraction was performed by removing the signal coming from the whole helix except the masked region for local refinement (*52*). We used the map generated by RELION 3D refinement to create a mask with UCSF ChimeraX (*53*) containing the three central protomers. The reference model was generated by 3D reconstruction, because of previous alignment parameters from 3D refinement, and used for 3D classification and, finally, local 3D refinement. The subtraction was performed on both strands and combined after particle rotation to reach the final 5.3-Å resolution with local refinement on Cryosparc. All the resulting maps from the 3D refinement protocol were postprocessed using DeepEMhancer (*54*), and local resolution maps were calculated using CryoSPARC v3.3.2.

### Pseudo-atomic model building

To build the atomic model, the crystal structure (PDB code: 5TJW) was docked in the focused reconstruction map by rigid body using UCSF ChimeraX as a starting reference and then refined using Coot (*55*) and validated with Phenix (*56*). The model was then fitted in the outer protomers NP^−1^ and NP^+1^ of the focused reconstruction map without further adjustments given that the resolution in these areas does not permit further interpretation. Of note, the oligomerization loop of the NP^+1^ protomer was kept in the model, although there is no density associated in the focused reconstruction map due to the mask used during signal subtraction. The phosphodiester backbone of the RNA molecule was modeled in density with an arbitrary 5′-3′ given orientation, and the bases were omitted.

The RNP-like helical pseudo-atomic model was generated by applying a C2 symmetry with helical parameters such as 24.3-Å rise and 57.4° twist on the NP model. The antiparallel helical model was generated using UCSF ChimeraX by applying a D2 symmetry on one strand of the RNP-like structure. The final cryo-EM reconstructions and fitted coordinates were deposited with the EM data bank under accession codes EMD-18043 and EMD-18044, with the associated PDB codes 8PZP and 8PZQ, respectively.

## Supplementary Materials

### This PDF file includes:

Figs. S1 to S10 Tables S1 to S3 Legend for movie S1 References
