# Supplementary material for: Cryo-EM structure of influenza helical nucleocapsid reveals NP-NP and NP-RNA interactions as a model for the genome encapsidation
Source: Sci Adv. 2023 Dec 15;9(50):eadj9974. doi: 10.1126/sciadv.adj9974 (PMC10848707; doi:10.1126/sciadv.adj9974)
Supplement: Supplementary file 1 — Figs. S1 to S10 Tables S1 to S3 Legend for movie S1 References [file sciadv.adj9974_sm.pdf]

Science Advances
AAAS

Supplementary Materials for

Cryo-EM structure of influenza helical nucleocapsid reveals NP-NP and NP-RNA interactions as a model for the genome encapsidation

Florian Chenavier *et al.*

Corresponding author: Thibaut Crépin, thibaut.crepin@ibs.fr; Allison Ballandras-Colas, allison.ballandras-colas@ibs.fr

Sci. Adv. **9**, eadj9974 (2023)
DOI: 10.1126/sciadv.adj9974

The PDF file includes:

Figs. S1 to S10
Tables S1 to S3
Legend for movie S1
References

Other Supplementary Material for this manuscript includes the following:

Movie S1

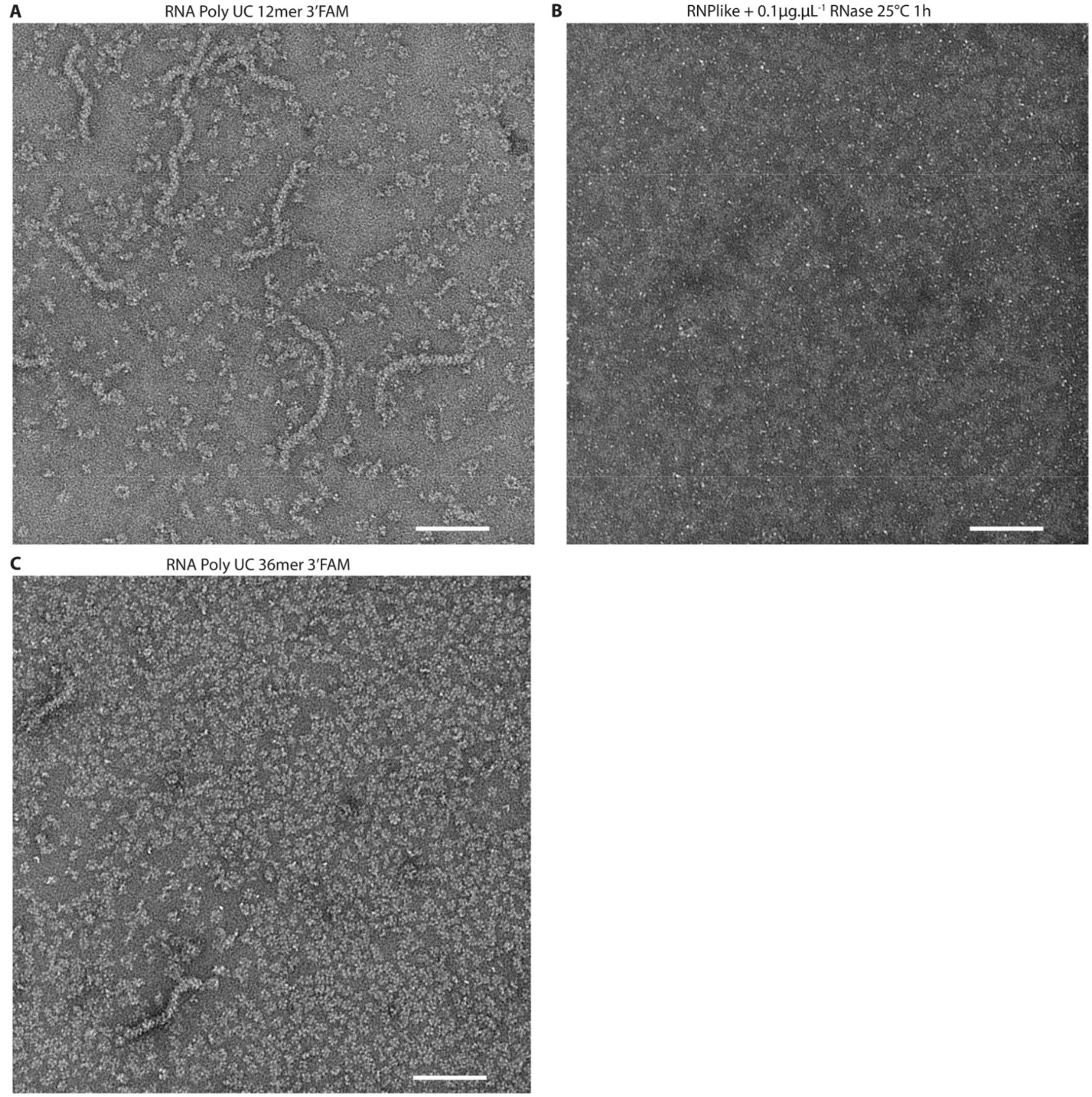

Figure S1. Micrographs of negatively stained particles. Visualization and comparison of the particles formed when the RNP-like assembled with the $^{5'}$P-(UC)$_6$-FAM$^{3'}$ (**A**), or the $^{5'}$P-(UC)$_6$-FAM$^{3'}$ followed by 1h treatment with RNase 0.1 µg.µL-1 (**B**), or assembled with $^{5'}$P-(UC)$_{18}$-FAM$^{3'}$ (**C**). All assembly reactions were carried out with the same amount of initial material to allow fair comparison of resulting RNP-like formed. Scale bar indicates 100 nm.

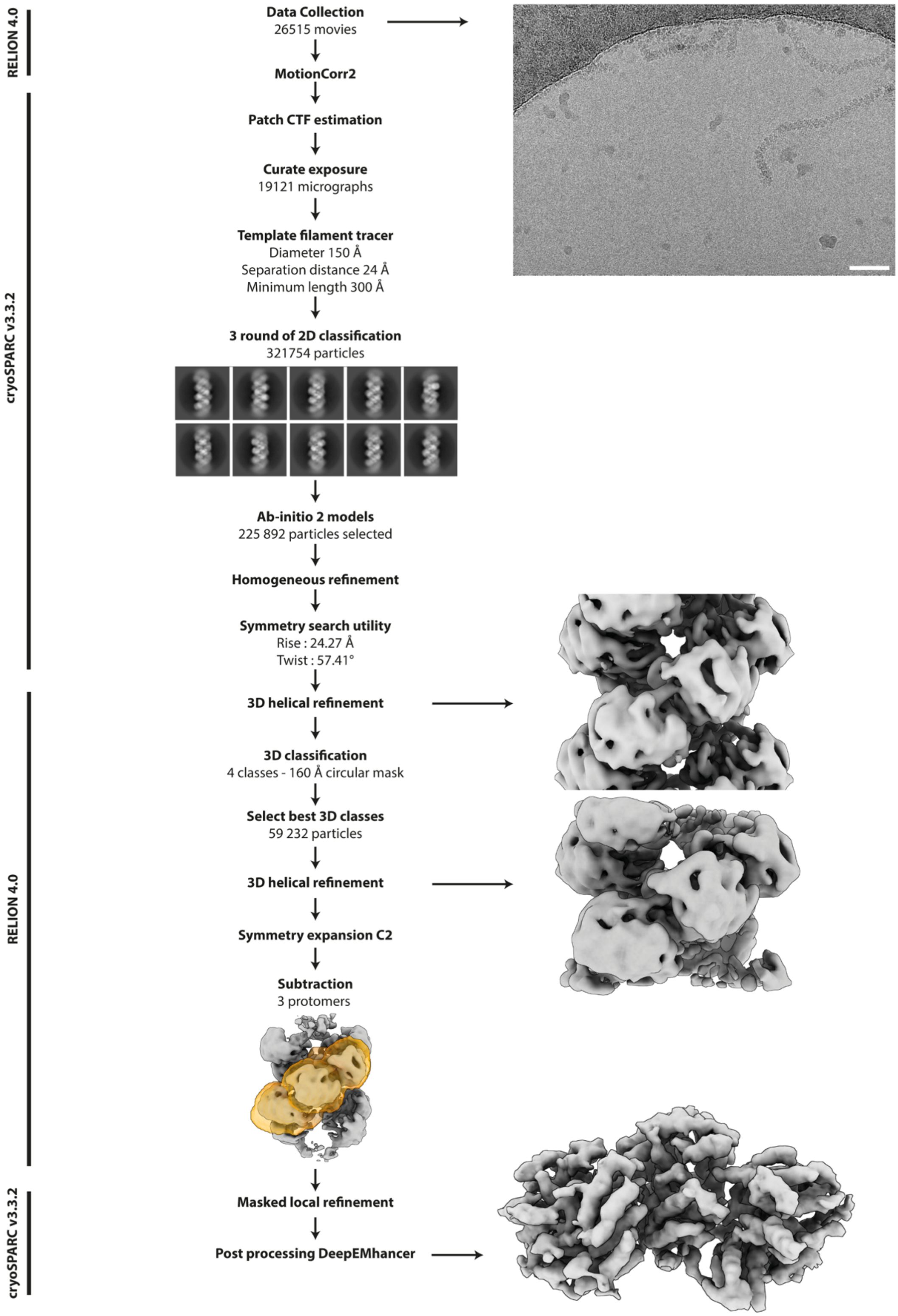

Figure S2. Cryo-EM data analysis workflow. Data processing steps were performed in Relion 4.0 (*50, 51*) and CryoSPARC v3.3.2 (*48*) and detailed in the Materials and Methods section. Analysis produced two distinct EM maps from the same data set, one of the helical RNP-like particles, the other corresponding to the local reconstruction post signal subtraction. Scale bar for the micrograph is 500 Å.

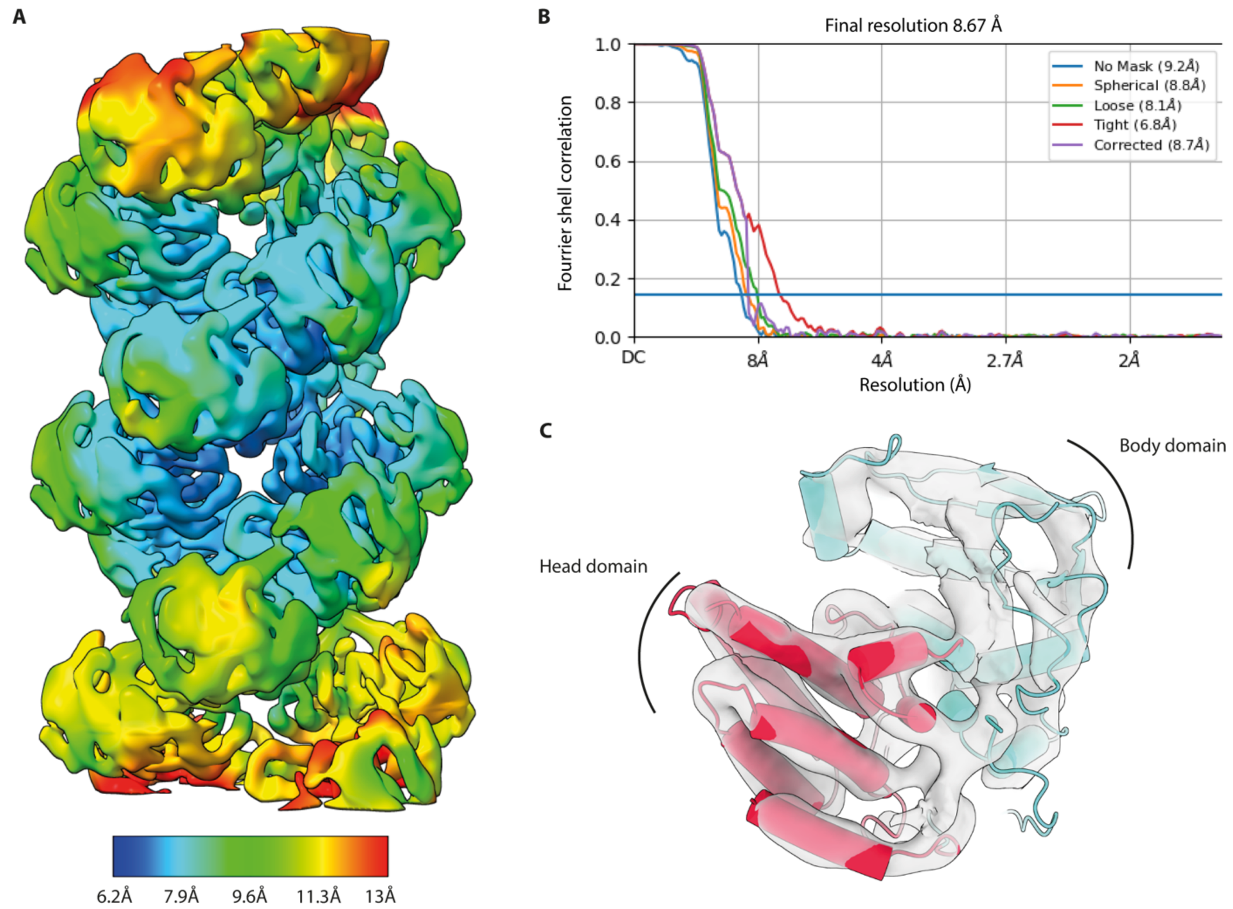

Figure S3. Analysis and validation of the helical reconstruction. A, cryo-EM map colored by local resolution estimated in CryoSPARC. **B**, Fourier shell correlation (FSC) curves for half-maps, with FSC cutoffs 0.143 indicated by the horizontal blue line (top right). **C**, NP crystal structure (PDB code: 5TJW) fitted to map as a rigid body, head domain made of helix bundle colored in red, body domain in cyan.

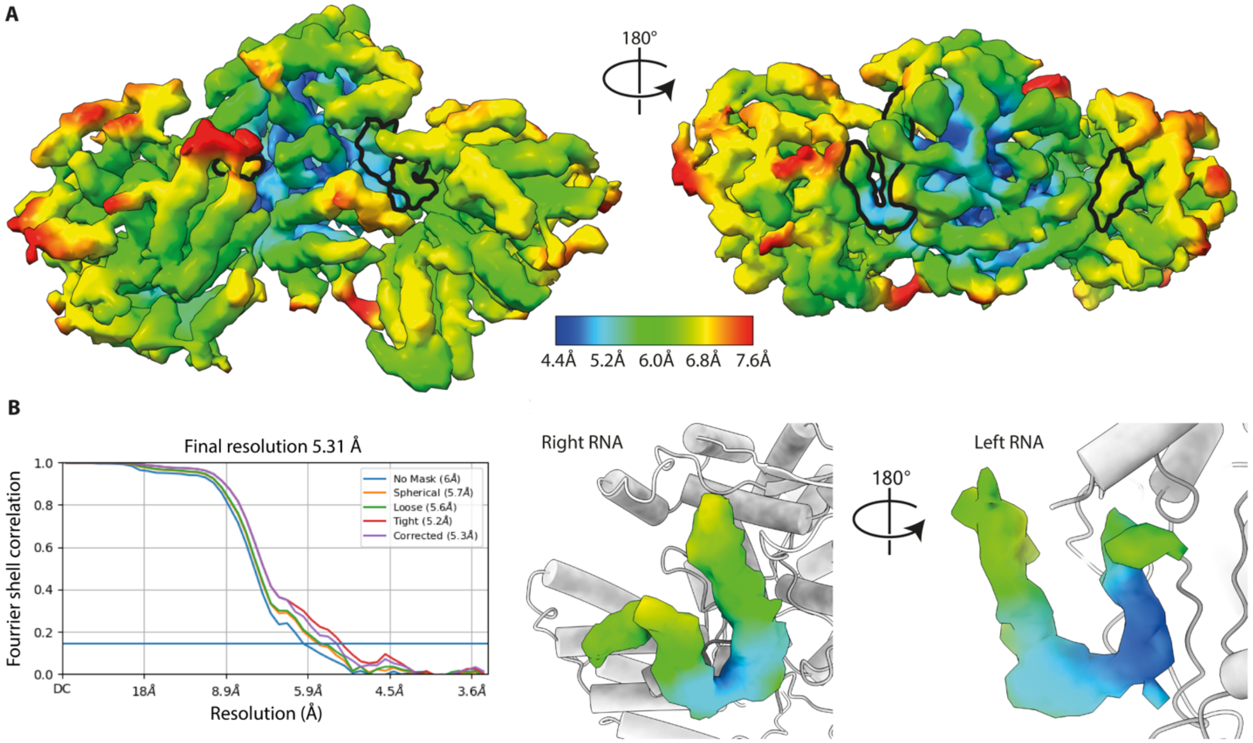

Figure S4. Analysis and validation of the local reconstruction. A, cryo-EM map colored by local resolution estimated in CryoSPARC. Density corresponding to the RNA is highlighted with a black outline. **B**, Fourier shell correlation (FSC) curves for half-maps, with FSC cutoff 0.143 indicated by the horizontal blue line. **C**, RNA segmented map on top of NP grey cartoon colored by local resolution according to scale in panel A.

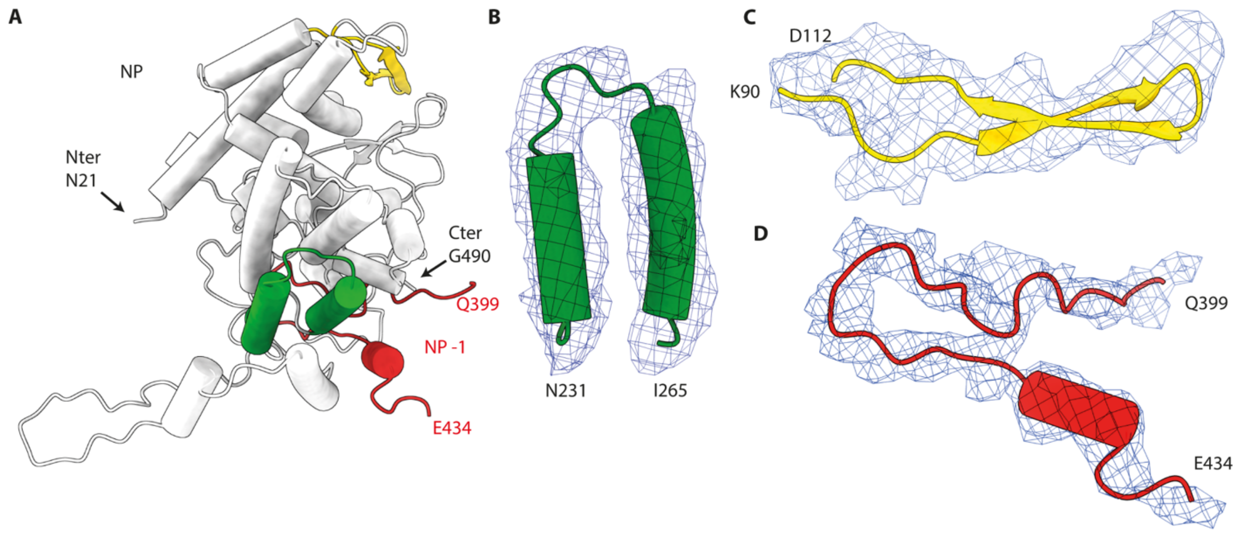

Figure S5. Refined atomic model. NP protomer is in white cartoon (**A**) with region of specific interest highlighted in green, yellow and red corresponding to two α-helices (**B**), a β-sheet (**C**) and the oligomerization loop from neighboring NP protomer (**D**) respectively with the cryo-EM densities shown as meshes.

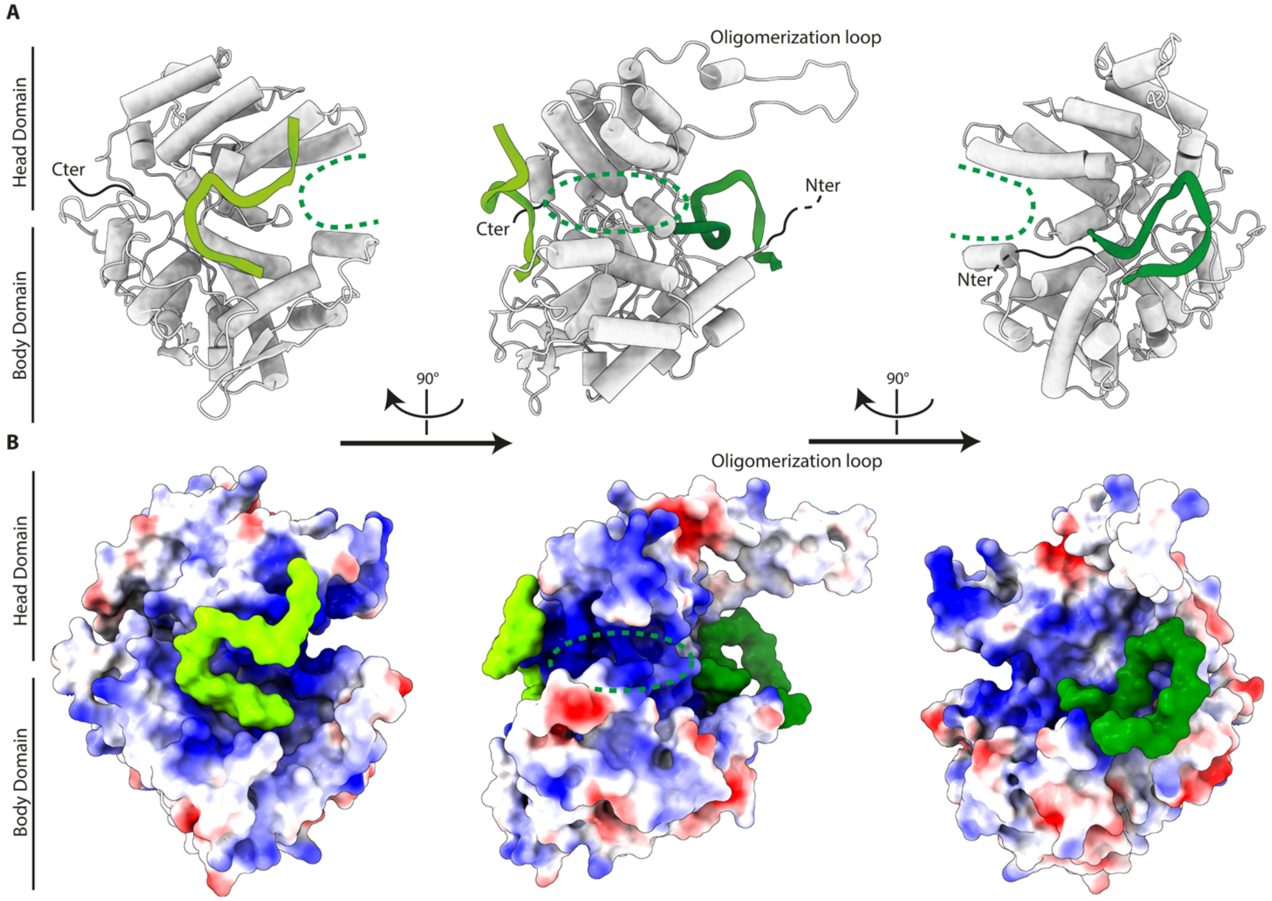

Figure S6. NP topology. This figure defines the terminology used in the manuscript. Three orientations (turned by 90°) of IAV NP are shown in (**A**) cartoon or in (**B**) electrostatic representation. NP is made by a head and a body domain. The hinge between the two domains forms a positively charged central binding groove (green dotted line) that has been previously described to accommodate RNA, suggesting that the right (forest) and left (chartreuse) RNA observed in our structure could be connected through that groove.

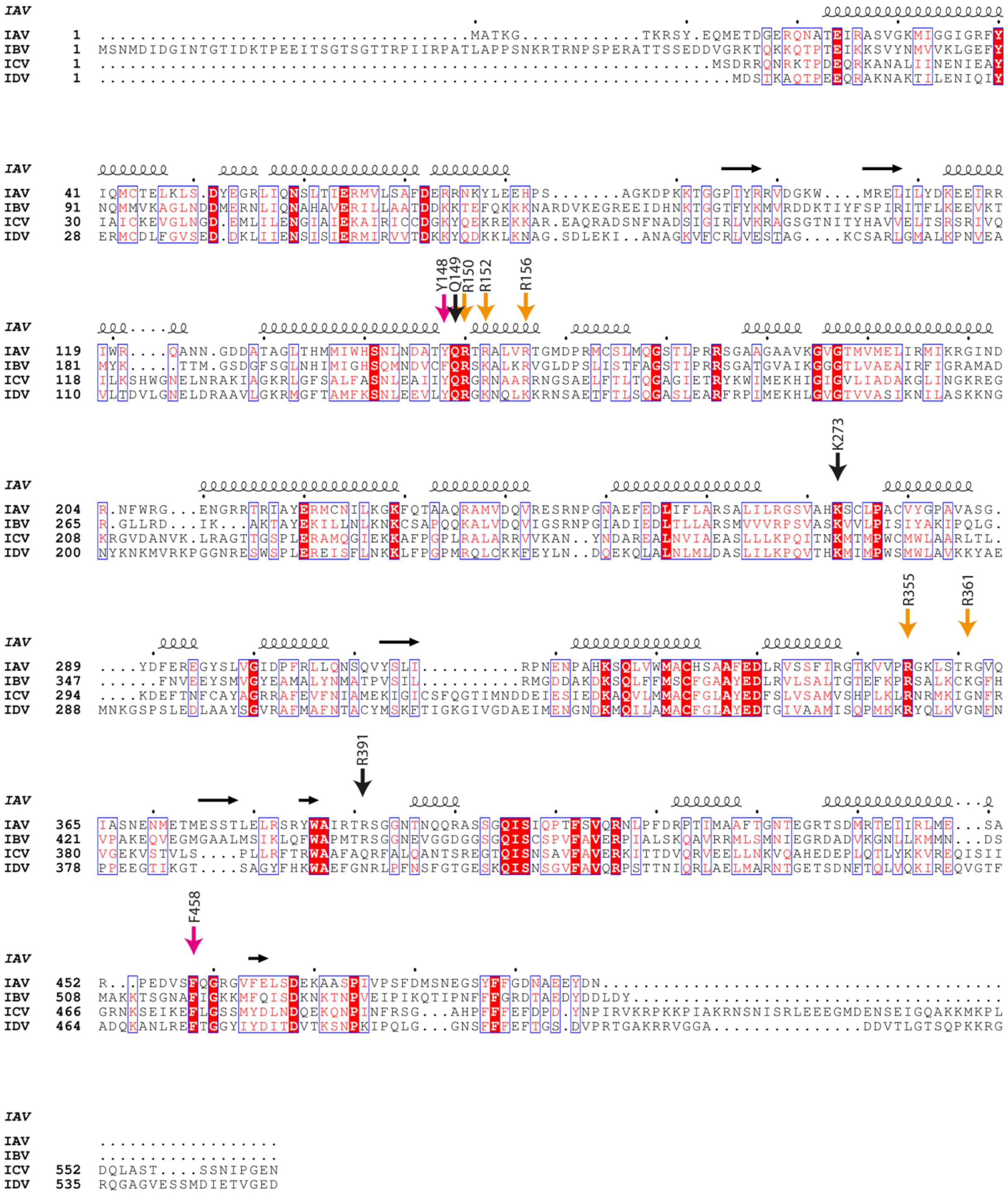

Figure S7. Primary NP sequence alignment from representative strains of influenza A (A/WSN/1933), B (B/Managua/4577-01/2008), C (C/California/1978) and D (D/bovine/France/2986/2012) virus. Arrows indicate the residues involved in RNA binding (*18-20,57*), colors correspond to code used in Figure 3. Figure generated with ESPript 3.0 (*58*).

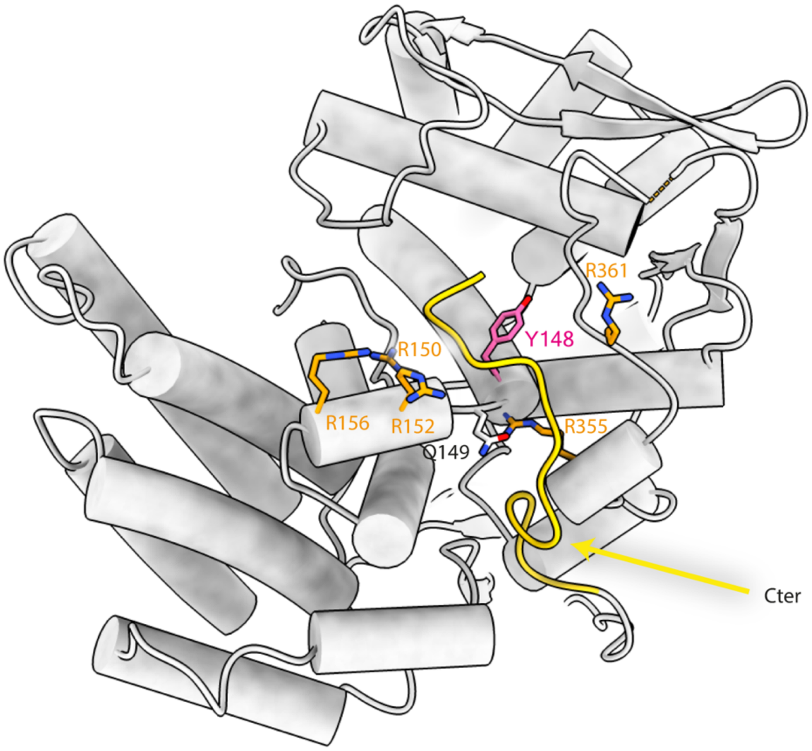

Figure S8. Highlights of the residues binding the C-terminal tail of NP_{R416A} (white cartoon). Residues of the C-terminal tail are shown in yellow cartoon, interacting residues from the head and body domain are display in orange, pink and white sticks according to the color code used in **Figure 3**.

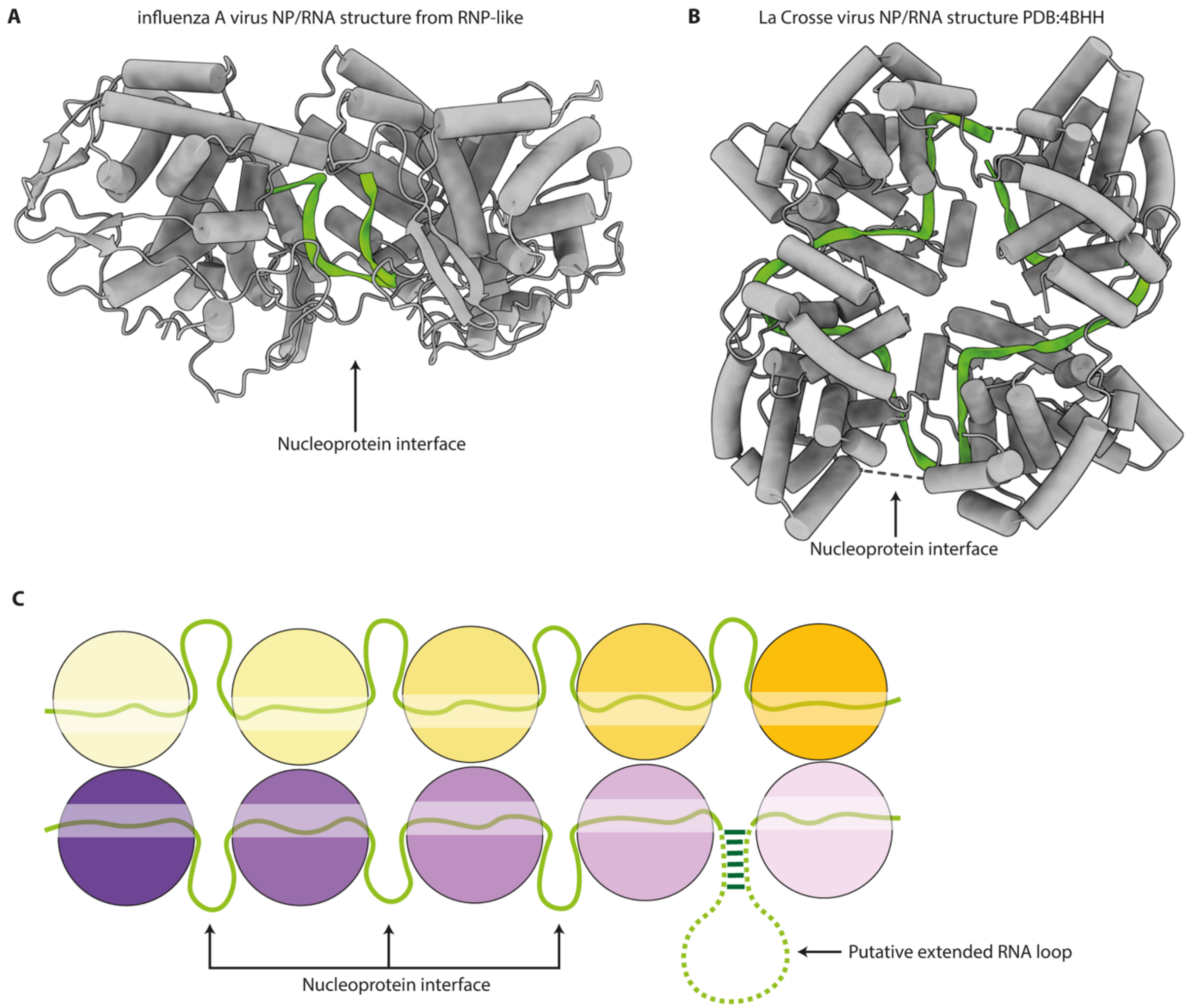

Figure S9. Comparison of IAV NP-RNA interaction (A) with known structure of La Crosse virus NP-RNA conformation (B). Proteins are shown in grey cartoon and RNA backbone in green ribbon. **C**, Schematic model of the RNA pathway for RNP of influenza A virus and La Crosse virus, showing binding of the RNA (green line) in the basic groove (light areas) and interaction at the NP-NP interface. Each strand is colored with either a white-to-purple or a white-to-yellow color gradient. The dotted green loop indicates a putative extended RNA for assembly of the RNPs during virus budding.

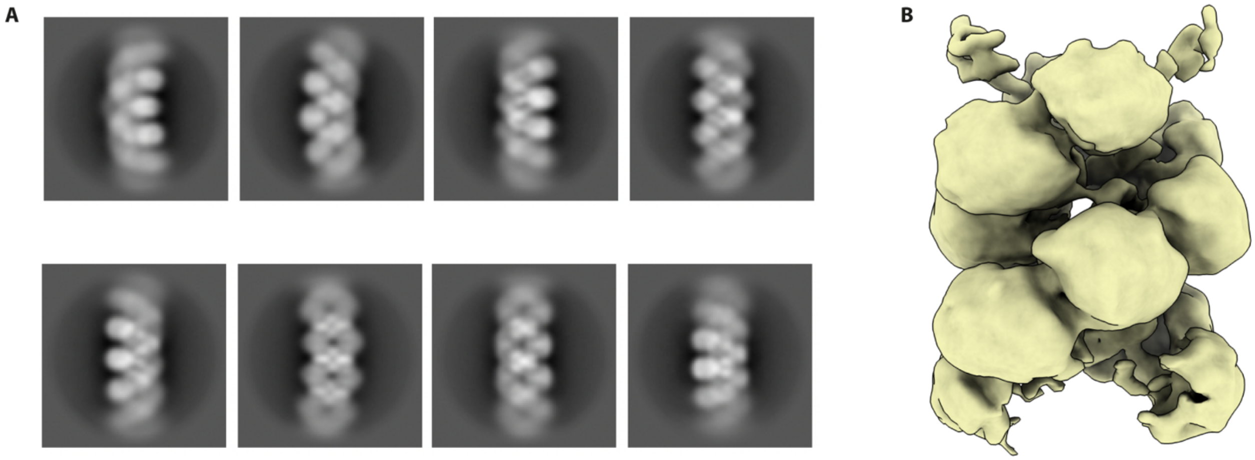

Figure S10. Analysis of the cryo-EM data collected on the Glacios microscope. The analysis was performed using CryoSPARC v3.3.2 (*48*) on 2,341 images. (A) Final 2D classes. (B) *Ab-initio* reconstruction at 15 Å resolution.

Table S1. Comparison of different RNA sequences affinity for NP. Anisotropy fluorescence measurements comparing different RNA sequences in two different buffers (20 mM Tris-HCl pH 7.5, 5 mM β-mercaptoethanol, 50 mM or 150 mM NaCl). Experiment was done in triplicates, standard deviation indicated next to measured value.

RNA molecules	50 mM NaCl	150 mM NaCl
$^{5'}$P-A_{12}-FAM	34.6 ± 4.7 nM	170.8 ± 16.2 nM
$^{5'}$P-U_{12}-FAM	13.1 ± 1.9 nM	120.2 ± 5.3 nM
$^{5'}$P-C_{12}-FAM	41.6 ± 3.3 nM	331.6 ± 24.6 nM
$^{5'}$P-$(UC)_6$-FAM	13.5 ± 1.9 nM	57.1 ± 2.5 nM

Table S2. Cryo-EM data collection, refinement and validation statistics.

**Data Collection and Processing**	**Helical reconstruction**	**Local reconstruction**
Microscope	Titan Krios	
Voltage (kV)	300	
Camera	Gatan K3-Summit	
Energy filter	Gatan Quantum-LS	
Magnification	105k	
Pixel size (Å)	0.84	
Defocus range (μm)	-2.2 to -0.8	
Electron exposure (e^{-1}/ Å^{-2})	40.8	
Number of good micrographs	19,121	
Initial number of particles	2,074,258	
Final number of particles	225,892	59,232
Symmetry imposed	Helical / C2	NA
Rise (Å) / twist (°)	24.27 / 57.41	
Map resolution (Å)	8.7	5.3
FSC threshold	0.143	0.143
Map resolution range (Å)	6.5-8.7	4.4-7.6
**Coordinate and B-factor refinement**		
Model resolution (Å)	NA	6.1
FSC threshold		0.143
Model resolution range (Å)	NA	5.5 - ∞
Map sharpening B-factor (Å^2)	-920.3	-381.9
Model composition	NA	
Number of atoms (non-H)		3,980
Protein residues		470
Ligands (nucleotides)		22
B-factors (Å^2)	NA	
Mean B-factor protein atoms (Å^2)		150.6
Mean B-factor of non-protein atoms (Å^2)		179.9
R.M.S deviations	NA	
RMSD bonds (Å)		0.004
RMSD bond angles (°)		1.091
**Ramachandran plot**		
Favored (%)	NA	95.30
Allowed (%)	NA	4.70
Disallowed (%)	NA	0
**Validation**		
Molprobity score	NA	2.09
All-atom clashscore	NA	18.31
Rotamer outliers (%)	NA	0

Table S3. NP mutation effects on virus viability and NP-RNA binding affinity (original paper cited in the table).

**NP mutation**	**Generation of mutant virus using reverse genetic** (*59*)	**NP mutants affinity for RNA, measured by Surface Plasmon Resonance**
Y148A	Non-viable virus	7-fold less than WT (*18*)
Q149A	Virus attenuated by more than 1 log unit compared to WT virus	
R150A	Non-viable virus	7.5-fold less than WT (*18*)
R152A	Non-viable virus	7.5-fold less than WT (*18*)
R156A	Non-viable virus	6-fold less than WT (*18*)
K273A	Non-viable virus	
R355A	Non-viable virus	
R361A	Non-viable virus	3-fold less than WT (*23*)
R391A	Virus attenuated by more than 2 log units compared to WT virus	

Movie caption

Movie 1: Overview of the RNP-like particle cryo-EM reconstruction. The movie summarizes the results presented in the article, using the same colors used in the different figures.

REFERENCES AND NOTES

1. D. McGeoch, P. Fellner, C. Newton, Influenza virus genome consists of eight distinct RNA species. *Proc. Natl. Acad. Sci. U.S.A.* **73**, 3045–3049 (1976).

2. A. J. Eisfeld, G. Neumann, Y. Kawaoka, At the centre: Influenza A virus ribonucleoproteins. *Nat. Rev. Microbiol.* **13**, 28–41 (2015).

3. S. R. Matzinger, T. D. Carroll, J. C. Dutra, Z.-M. Ma, C. J. Miller, Myxovirus resistance gene A (MxA) expression suppresses influenza A virus replication in alpha interferon-treated primate cells. *J. Virol.* **87**, 1150–1158 (2013).

4. B. Mänz, D. Dornfeld, V. Götz, R. Zell, P. Zimmermann, O. Haller, G. Kochs, M. Schwemmle, Pandemic influenza a viruses escape from restriction by human mxa through adaptive mutations in the nucleoprotein. *PLOS Pathog.* **9**, e1003279 (2013).

5. R. M. Pinto, S. Bakshi, S. Lytras, M. K. Zakaria, S. Swingler, J. C. Worrell, V. Herder, K. E. Hargrave, M. Varjak, N. Cameron-Ruiz, M. Collados Rodriguez, M. Varela, A. Wickenhagen, C. Loney, Y. Pei, J. Hughes, E. Valette, M. L. Turnbull, W. Furnon, Q. Gu, L. Orr, A. Taggart, O. Diebold, C. Davis, C. Boutell, F. Grey, E. Hutchinson, P. Digard, I. Monne, S. K. Wootton, M. K. L. MacLeod, S. J. Wilson, M. Palmarini, BTN3A3 evasion promotes the zoonotic potential of influenza A viruses. *Nature*, **619**, 338–347 (2023).

6. M. W. Pons, I. T. Schulze, G. K. Hirst, R. Hauser, Isolation and characterization of the ribonucleoprotein of influenza virus. *Virology* **39**, 250–259 (1969).

7. R. W. Compans, J. Content, P. H. Duesberg, Structure of the ribonucleoprotein of influenza virus. *J. Virol.* **10**, 795–800 (1972).

8. R. Arranz, R. Coloma, F. J. Chichón, J. J. Conesa, J. L. Carrascosa, J. M. Valpuesta, J. Ortín, J. Martín-Benito, The structure of native influenza virion ribonucleoproteins. *Science* **338**, 1634–1637 (2012).

9. A. Moeller, R. N. Kirchdoerfer, C. S. Potter, B. Carragher, I. A. Wilson, Organization of the influenza virus replication machinery. *Science* **338**, 1631–1634 (2012).

10. R. Coloma, R. Arranz, J. M. de la Rosa-Trevín, C. O. S. Sorzano, S. Munier, D. Carlero, N. Naffakh, J. Ortín, J. Martín-Benito, Structural insights into influenza A virus ribonucleoproteins reveal a processive helical track as transcription mechanism. *Nat. Microbiol.* **5**, 727–734 (2020).

11. Q. Ye, R. M. Krug, Y. J. Tao, The mechanism by which influenza A virus nucleoprotein forms oligomers and binds RNA. *Nature* **444**, 1078–1082 (2006).

12. R. W. Ruigrok, F. Baudin, Structure of influenza virus ribonucleoprotein particles. II. Purified RNA-free influenza virus ribonucleoprotein forms structures that are indistinguishable from the intact influenza virus ribonucleoprotein particles. *J. Gen. Virol.* **76**, 1009–1014 (1995).

13. A. Labaronne, C. Swale, A. Monod, G. Schoehn, T. Crépin, R. W. H. Ruigrok, Binding of RNA by the nucleoproteins of influenza viruses A and B. *Viruses* **8**, 247 (2016).

14. J. R. Gallagher, U. Torian, D. M. McCraw, A. K. Harris, Structural studies of influenza virus RNPs by electron microscopy indicate molecular contortions within NP supra-structures. *J. Struct. Biol.* **197**, 294–307 (2017).

15. S. Lynch, D. Kolakofsky, Ends of the RNA within Sendai virus defective interfering nucleocapsids are not free. *J. Virol.* **28**, 584–589 (1978).

16. Z. Zhang, H. Shigematsu, T. Shimizu, U. Ohto, Improving particle quality in cryo-EM analysis using a PEGylation method. *Structure* **29**, 1192–1199 (2021).

17. M. Nakano, Y. Sugita, N. Kodera, S. Miyamoto, Y. Muramoto, M. Wolf, T. Noda, Ultrastructure of influenza virus ribonucleoprotein complexes during viral RNA synthesis. *Commun. Biol.* **4**, 858 (2021).

18. C.-L. Liu, H.-C. Hung, S.-C. Lo, C.-H. Chiang, I.-J. Chen, J. T.-A. Hsu, M.-H. Hou, Using mutagenesis to explore conserved residues in the RNA-binding groove of influenza A virus nucleoprotein for antiviral drug development. *Sci. Rep.* **6**, 21662 (2016).

19. Y.-S. Tang, S. Xu, Y.-W. Chen, J.-H. Wang, P.-C. Shaw, Crystal structures of influenza nucleoprotein complexed with nucleic acid provide insights into the mechanism of RNA interaction. *Nucleic Acids Res.* **49**, 4144–4154 (2021).

20. S. Chenavas, L. F. Estrozi, A. Slama-Schwok, B. Delmas, C. Di Primo, F. Baudin, X. Li, T. Crépin, R. W. H. Ruigrok, Monomeric nucleoprotein of influenza A virus. *PLOS Pathog.* **9**, e1003275 (2013).

21. L. Hanke, K. E. Knockenhauer, R. C. Brewer, E. van Diest, F. I. Schmidt, T. U. Schwartz, H. L. Ploegh, The antiviral mechanism of an influenza A virus nucleoprotein-specific single-domain antibody fragment. *MBio* **7**, e01569-01516 (2016).

22. B. Pang, N. N. Cheung, W. Zhang, J. Dai, R. Y. Kao, H. Zhang, Q. Hao, Structural characterization of H1N1 nucleoprotein-nucleozin binding sites. *Sci. Rep.* **6**, 29684 (2016).

23. B. Tarus, C. Chevalier, C.-A. Richard, B. Delmas, C. Di Primo, A. Slama-Schwok, Molecular dynamics studies of the nucleoprotein of influenza A virus: Role of the protein flexibility in RNA binding. *PLOS ONE* **7**, e30038 (2012).

24. A. Guillon, D. Brea-Diakite, A. Cezard, A. Wacquiez, T. Baranek, J. Bourgeais, F. Picou, V. Vasseur, L. Meyer, C. Chevalier, A. Auvet, J. M. Carballido, L. Nadal Desbarats, F. Dingli, A. Turtoi, A. Le Gouellec, F. Fauvelle, A. Donchet, T. Crépin, P. S. Hiemstra, C. Paget, D. Loew, O. Herault, N. Naffakh, R. Le Goffic, M. Si-Tahar, Host succinate inhibits influenza virus infection through succinylation and nuclear retention of the viral nucleoprotein. *EMBO J.* **41**, e108306 (2022).

25. E. C. Hutchinson, P. D. Charles, S. S. Hester, B. Thomas, D. Trudgian, M. Martínez-Alonso, E. Fodor, Conserved and host-specific features of influenza virion architecture. *Nat. Commun.* **5**, 4816 (2014).

26. J. Ortega, J. Martín-Benito, T. Zürcher, J. M. Valpuesta, J. L. Carrascosa, J. Ortín, Ultrastructural and functional analyses of recombinant influenza virus ribonucleoproteins suggest dimerization of nucleoprotein during virus amplification. *J. Virol.* **74**, 156–163 (2000).

27. B. Dadonaite, B. Gilbertson, M. L. Knight, S. Trifkovic, S. Rockman, A. Laederach, L. E. Brown, E. Fodor, D. L. V. Bauer, The structure of the influenza A virus genome. *Nat. Microbiol.* **4**, 1781–1789 (2019).

28. C. Jakob, R. Paul-Stansilaus, M. Schwemmle, R. Marquet, H. Bolte, The influenza A virus genome packaging network—Complex, flexible and yet unsolved. *Nucleic Acids Res.* **50**, 9023–9038 (2022).

29. A. Modrego, D. Carlero, R. Arranz, J. Martín-Benito, CryoEM of viral ribonucleoproteins and nucleocapsids of single-stranded RNA viruses. *Viruses* **15**, 653 (2023).

30. A. Ariza, S. J. Tanner, C. T. Walter, K. C. Dent, D. A. Shepherd, W. Wu, S. V. Matthews, J. A. Hiscox, T. J. Green, M. Luo, R. M. Elliott, A. R. Fooks, A. E. Ashcroft, N. J. Stonehouse, N. A. Ranson, J. N. Barr, T. A. Edwards, Nucleocapsid protein structures from orthobunyaviruses reveal insight into ribonucleoprotein architecture and RNA polymerization. *Nucleic Acids Res.* **41**, 5912–5926 (2013).

31. J. Reguera, H. Malet, F. Weber, S. Cusack, Structural basis for encapsidation of genomic RNA by La Crosse Orthobunyavirus nucleoprotein. *Proc. Natl. Acad. Sci. U.S.A.* **110**, 7246–7251 (2013).

32. D. D. Raymond, M. E. Piper, S. R. Gerrard, G. Skiniotis, J. L. Smith, Phleboviruses encapsidate their genomes by sequestering RNA bases. *Proc. Natl. Acad. Sci.* **109**, 19208–19213 (2012).

33. F. R. Hopkins, B. Álvarez-Rodríguez, G. R. Heath, K. Panayi, S. Hover, T. A. Edwards, J. N. Barr, J. Fontana, The native orthobunyavirus ribonucleoprotein possesses a helical architecture. *MBio* **13**, e0140522 (2022).

34. S. Milles, M. R. Jensen, G. Communie, D. Maurin, G. Schoehn, R. W. H. Ruigrok, M. Blackledge, Self-assembly of measles virus nucleocapsid-like particles: Kinetics and RNA sequence dependence. *Angew. Chem. Int. Ed. Engl.* **55**, 9356–9360 (2016).

35. G. Schoehn, M. Mavrakis, A. Albertini, R. Wade, A. Hoenger, R. W. H. Ruigrok, The 12 Å structure of trypsin-treated measles virus N–RNA. *J. Mol. Biol.* **339**, 301–312 (2004).

36. I. Gutsche, A. Desfosses, G. Effantin, W. L. Ling, M. Haupt, R. W. H. Ruigrok, C. Sachse, G. Schoehn, Near-atomic cryo-EM structure of the helical measles virus nucleocapsid. *Science* **348**, 704–707 (2015).

37. A. R. Camacho-Zarco, L. Yu, T. Krischuns, S. Dedeoglu, D. Maurin, G. Bouvignies, T. Crépin, R. W. H. Ruigrok, S. Cusack, N. Naffakh, M. Blackledge, Multivalent dynamic colocalization of avian influenza polymerase and nucleoprotein by intrinsically disordered ANP32A reveals the molecular basis of human adaptation. *J. Am. Chem. Soc.* **145**, 20985–21001 (2023).

38. F. Baudin, I. Petit, W. Weissenhorn, R. W. Ruigrok, *In vitro* dissection of the membrane and RNP binding activities of influenza virus M1 protein. *Virology* **281**, 102–108 (2001).

39. K. Klumpp, R. W. Ruigrok, F. Baudin, Roles of the influenza virus polymerase and nucleoprotein in forming a functional RNP structure. *EMBO J.* **16**, 1248–1257 (1997).

40. I. T. Schulze, Structure of the influenza virion. *Adv. Virus Res.* **18**, 1–55 (1973).

41. P. A. Jennings, J. T. Finch, G. Winter, J. S. Robertson, Does the higher order structure of the influenza virus ribonucleoprotein guide sequence rearrangements in influenza viral RNA? *Cell* **34**, 619–627 (1983).

42. D. Nečas, P. Klapetek, Gwyddion: An open-source software for SPM data analysis. *Centr. Eur. J.Phys.* **10**, 181–188 (2012).

43. S.-w. W. Chen, J.-L. Pellequer, DeStripe: Frequency-based algorithm for removing stripe noises from AFM images. *BMC Struct. Biol.* **11**, 7 (2011).

44. S.-w. W. Chen, J.-M. Teulon, C. Godon, J.-L. Pellequer, Atomic force microscope, molecular imaging, and analysis. *J. Mol. Recognit.* **29**, 51–55 (2016).

45. S.-W. W. Chen, A.-S. Banneville, J.-M. Teulon, J. Timmins, J.-L. Pellequer, Nanoscale surface structures of DNA bound to Deinococcus radiodurans HU unveiled by atomic force microscopy. *Nanoscale* **12**, 22628–22638 (2020).

46. E. Kandiah, T. Giraud, A. de Maria Antolinos, F. Dobias, G. Effantin, D. Flot, M. Hons, G. Schoehn, J. Susini, O. Svensson, G. A. Leonard, C. Mueller-Dieckmann, CM01: A facility for cryo-electron microscopy at the european synchrotron. *Acta Crystallogr. D Struct. Biol.* **75**, 528–535 (2019).

47. X. Li, P. Mooney, S. Zheng, C. R. Booth, M. B. Braunfeld, S. Gubbens, D. A. Agard, Y. Cheng, Electron counting and beam-induced motion correction enable near-atomic-resolution single-particle cryo-EM. *Nat. Methods* **10**, 584–590 (2013).

48. A. Punjani, J. L. Rubinstein, D. J. Fleet, M. A. Brubaker, cryoSPARC: Algorithms for rapid unsupervised cryo-EM structure determination. *Nat. Methods* **14**, 290–296 (2017).

49. R. Fernandez-Leiro, S. Scheres, A pipeline approach to single-particle processing in RELION. *Acta Crystallogr. D Struct. Biol.* **73**, 496–502 (2017).

50. S. He, S. H. W. Scheres, Helical reconstruction in RELION. *J. Struct. Biol.* **198**, 163–176 (2017).

51. D. Kimanius, L. Dong, G. Sharov, T. Nakane, S. H. W. Scheres, New tools for automated cryo-EM single-particle analysis in RELION-4.0. *Biochem. J.* **478**, 4169–4185 (2021).

52. C. Bayly-Jones, C. J. Lupton, C. Fritz, H. Venugopal, D. Ramsbeck, M. Wermann, C. Jäger, A. de Marco, S. Schilling, D. Schlenzig, J. C. Whisstock, Helical ultrastructure of the metalloprotease meprin α in complex with a small molecule inhibitor. *Nat. Commun.* **13**, 6178 (2022).

53. E. F. Pettersen, T. D. Goddard, C. C. Huang, E. C. Meng, G. S. Couch, T. I. Croll, J. H. Morris, T. E. Ferrin, UCSF ChimeraX structure visualization for researchers, educators, and developers. *Protein Sci.* **30**, 70–82 (2021).

54. R. Sanchez-Garcia, J. Gomez-Blanco, A. Cuervo, J. M. Carazo, C. O. S. Sorzano, J. Vargas, DeepEMhancer: A deep learning solution for cryo-EM volume post-processing. *Commun. Biol.* **4**, 874 (2021).

55. P. Emsley, B. Lohkamp, W. G. Scott, K. Cowtan, Features and development of Coot. *Acta Crystallogr. D Biol. Crystallogr.* **66**, 486–501 (2010).

56. V. B. Chen, W. B. Arendall, 3rd, J. J. Headd, D. A. Keedy, R. M. Immormino, G. J. Kapral, L. W. Murray, J. S. Richardson, D. C. Richardson, MolProbity: All-atom structure validation for macromolecular crystallography. *Acta Crystallogr. D Biol. Crystallogr.* **66**, 12–21 (2010).

57. E. C. Hutchinson, E. M. Denham, B. Thomas, D. C. Trudgian, S. S. Hester, G. Ridlova, A. York, L. Turrell, E. Fodor, Mapping the phosphoproteome of influenza A and B viruses by mass spectrometry. *PLOS Pathog.* **8**, e1002993 (2012).

58. X. Robert, P. Gouet, Deciphering key features in protein structures with the new ENDscript server. *Nucleic Acids Res.* **42**, W320–W324 (2014).

59. Z. Li, T. Watanabe, M. Hatta, S. Watanabe, A. Nanbo, M. Ozawa, S. Kakugawa, M. Shimojima, S. Yamada, G. Neumann, Y. Kawaoka, Mutational analysis of conserved amino acids in the influenza A virus nucleoprotein. *J. Virol.* **83**, 4153–4162 (2009).
